# Assessment of Neuronal Damage in Brain Slice Cultures Using Machine Learning Based on Spatial Features

**DOI:** 10.3389/fnins.2021.740178

**Published:** 2021-10-08

**Authors:** Urszula Hohmann, Faramarz Dehghani, Tim Hohmann

**Affiliations:** Department of Anatomy and Cell Biology, Martin Luther University Halle-Wittenberg, Halle (Saale), Germany

**Keywords:** machine learning, neuronal damage, slice culture, neural network, propidium iodide, image analysis

## Abstract

Neuronal damage presents a major health issue necessitating extensive research to identify mechanisms of neuronal cell death and potential therapeutic targets. Commonly used models are slice cultures out of different brain regions extracted from mice or rats, excitotoxically, ischemic, or traumatically lesioned and subsequently treated with potential neuroprotective agents. Thereby cell death is regularly assessed by measuring the propidium iodide (PI) uptake or counting of PI-positive nuclei. The applied methods have a limited applicability, either in terms of objectivity and time consumption or regarding its applicability. Consequently, new tools for analysis are needed. Here, we present a framework to mimic manual counting using machine learning algorithms as tools for semantic segmentation of PI-positive dead cells in hippocampal slice cultures. Therefore, we trained a support vector machine (SVM) to classify images into either “high” or “low” neuronal damage and used naïve Bayes, discriminant analysis, random forest, and a multilayer perceptron (MLP) as classifiers for segmentation of dead cells. In our final models, pixel-wise accuracies of up to 0.97 were achieved using the MLP classifier. Furthermore, a SVM-based post-processing step was introduced to differentiate between false-positive and false-negative detections using morphological features. As only very few false-positive objects and thus training data remained when using the final model, this approach only mildly improved the results. A final object splitting step using Hough transformations was used to account for overlap, leading to a recall of up to 97.6% of the manually assigned PI-positive dead cells. Taken together, we present an analysis tool that can help to objectively and reproducibly analyze neuronal damage in brain-derived slice cultures, taking advantage of the morphology of pycnotic cells for segmentation, object splitting, and identification of false positives.

## Introduction

Neurological disorders and traumata of the central nervous system are considered major public health issues. Currently, more than 2 million people are affected by traumatic brain injuries per year (Majdan et al., 2016). Based on this number, the taken efforts become comprehensible to better understand the molecular courses and to develop potential therapeutic strategies to limit neuronal damage after injury (Ladak et al., 2019).

Slice cultures are frequently used as model and represent the complex composition of neuronal tissue in a near *in vivo* manner. They can be prepared from different brain regions, but mostly the hippocampus is used (Thomas et al., 2007; Ebrahimi et al., 2010; Kim et al., 2014; Di Pietro et al., 2015; Li et al., 2016; Masuch et al., 2016a; Hohmann et al., 2019). In hippocampal slices, the neuronal cytoarchitecture and connections are well preserved, and from a single animal, up to six slice cultures can be obtained (Bahr, 1995; Dailey et al., 2013). The lack of spontaneous degeneration and simple accessibility are further arguments for the frequent use of this model to study neuronal damage (Dehghani et al., 2003; Hailer et al., 2005; Ebrahimi et al., 2010; Lee et al., 2011; Won et al., 2011; Pérez-Gómez and Tasker, 2012; Kim et al., 2014; Di Pietro et al., 2015; Masuch et al., 2016a,b; Grabiec et al., 2017, 2019; Grüßer et al., 2019; Hohmann et al., 2019). To assess the amount of neuronal damage, propidium iodide (PI) is used, in order to mark cellular death (Dehghani et al., 2003; Hailer et al., 2005; Ebrahimi et al., 2010; Lee et al., 2011; Su et al., 2011; Pérez-Gómez and Tasker, 2012; Kim et al., 2013, 2014; Di Pietro et al., 2015; Happ and Tasker, 2016; Masuch et al., 2016a; Grabiec et al., 2017, 2019; Grüßer et al., 2019; Hohmann et al., 2019). The evaluation of cell death in these cases is performed either by manually counting dead cells (Sun et al., 2009; Grabiec et al., 2017; Hohmann et al., 2019) or by cumulative intensity measures. Intensity measurements are performed in specified anatomical regions or *via* global thresholding and subsequently calculating the covered area or segmenting objects based on size and intensity (Hailer et al., 2005; Lee et al., 2011; Won et al., 2011; Pérez-Gómez and Tasker, 2012; Kim et al., 2013, 2014; Di Pietro et al., 2015; Happ and Tasker, 2016; Grüßer et al., 2019). Thresholding and cumulative intensity measurements suffer from very similar limitations in that they are poor metrics in noisy images. Furthermore, both methods intrinsically assume a very high specificity of the PI signal, which is often not given. The limited correlation of PI intensity and actual dead cells has been reported before (Ebrahimi et al., 2010). Additionally, when global thresholds were used, no algorithm was given on how to set this threshold, making it highly subjective and hard to reproduce. Manual counting on the other hand can adjust to noisy images, but lacks objectivity (limited inter-rater reliability) and is very time consuming. Consequently, more robust and evaluated analysis strategies are needed to evaluate neuronal damage in such conditions.

Here, we systematically tested four different machine learning classifiers, a naïve Bayes (NB) classifier, a discriminant analysis (DA) classifier, a random forest (RF) classifier, and a multilayer perceptron (MLP) classifier, to assess neuronal damage in hippocampal slice cultures. For the presented approach, we used spatial features that take advantage of the shape of PI-positive, degenerated nuclei. The results demonstrated that manual counting can very well be reproduced by the presented analysis scheme (maximal accuracy: 0.97) and thus is feasible for analyzing the extent of neuronal damage before and after specified interventions in form of the number of degenerated neurons.

## Materials and Methods

### Preparation and Labeling of Organotypic Hippocampal Slice Cultures

All animal experiments were performed in accordance with the Policy on Ethics and the Policy on the Use of Animals in Neuroscience Research as indicated in the directive 2010/63/EU of the European Parliament and of the Council of the European Union on the protection of animals used for scientific purposes and were approved by the local authorities for care and use of laboratory animals (State of Saxony-Anhalt, Germany, permission number: I11M18, date: 01.12.2012).

To prepare Organotypic Hippocampal Slice Cultures (OHSC), 5-day-old Bl6/J mice (Charles River, Sulzfeld, Germany) were decapitated and brains were dissected under aseptic conditions. After removal of the cerebellum and the frontal pole, the brains were placed in minimal essential medium (MEM, Invitrogen, Carlsbad, CA, United States), containing 1% (v/v) glutamine (Invitrogen) at 4°C. The brains were cut into 350-μm-thick slices with a sliding vibratome (Leica VT 1200 S, Leica Microsystems AG, Wetzlar, Germany). Three to five OHSC were obtained from each brain and immediately transferred into cell culture inserts (pore size 0.4 μm, Sarstedt, Nümbrecht, Germany). The cell culture inserts were then placed in six-well culture dishes (Greiner, Kremsmünster, Austria) containing 1-ml culture medium per well. The culture medium consisted of 50% (v/v) MEM, 25% (v/v) Hanks’ balanced salt solution (Invitrogen), 25% (v/v) normal horse serum (Invitrogen), 1% (v/v) glutamine (Invitrogen), 1.2 mg/ml glucose (Braun, Melsungen, Germany), and 1% (v/v) streptomycin/penicillin (Invitrogen) with a pH of 7.3. The OHSC in the culture dishes were incubated at 35°C in a fully humidified atmosphere with 5% CO_2_, and the cell culture medium was changed every second day.

On day 13, OHSC were lesioned with N-methyl-D-aspartic acid (NMDA, 10 μM, Sigma-Aldrich, St. Louis, MO, United States) for 4 h or left untreated. Slices of both groups were kept in culture medium for another 3 days.

### Staining and Imaging of Organotypic Hippocampal Slice Cultures

Two hours prior to fixation with 4% paraformaldehyde (Sigma-Aldrich), PI (5 μg/ml, Merck Millipore) was added to the culture medium. The OHSC were removed from the cell culture inserts, washed with phosphate buffered saline (PBS) containing 0.03% (v/v) Triton X-100 (Applichem, Darmstadt, Germany; PBS-T) for 10 min, following 5 min with Aqua dest, and mounted with DAKO fluorescent mounting medium (DAKO Diagnostika GmbH, Hamburg, Germany). Further analyses were performed with a CLSM (LSM 710 Meta, Zeiss). For detection of PI-labeled degenerating neurons, monochromatic light with a wavelength of 543 nm and an emission band pass filter for a wavelength of 585–615 nm were used. The dentate gyrus of the hippocampus was visualized with a 20× objective, as a z-stack with a step width of 2 μm. Resulting images had a resolution of 1024 × 1024 pixel and a pixel size of 0.52 μm.

### Semantic Classification of Neuronal Damage

All models and source codes were generated under Windows 10 using MATLAB R2021a with python’s sklearn and are available in the Supplementary Material and *via* GitHub: https://github.com/Herodot1/NeuronalDamage.git. The main output of the given framework is the number of degenerated, pycnotic nuclei in a given image stack. More detailed dependencies and how to use the framework are described in the manual supplied together with the source code.

**FIGURE 1:**
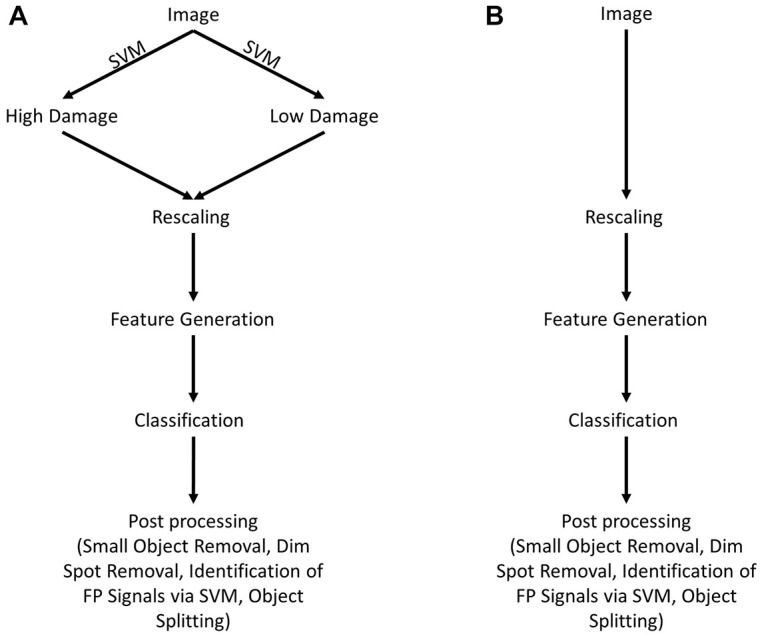
Scheme for image analysis. **(A)** As a first step, images were classified as either showing “high” or “low” neuronal damage using a support vector machine (SVM). Afterward, images are rescaled for reproducibility and features are generated. Depending on the type of image (“high” or “low” damage), the appropriate trained classifier is chosen and images are analyzed. As a last step, image post-processing is performed, including the removal of small (<30 pixels) and dim objects (<0.1 × maximal intensity). Furthermore, false-positive objects are identified by a trained SVM and removed as a consequence. **(B)** Shows an alternative way of image analysis that is almost identical to **(A)** without any differentiation between “high” and “low” damage images.

The following steps were conducted for acquisition and processing of data. A summary of the image analysis scheme is shown in Figure 1.

#### Acquisition of Training Data

An expert marked PI-positive (dead) cells. An h-maximum transform was then used to match manually marked positions to local maxima. True negative signals were defined using manually marked polygonal regions. Overall, 117 OHSC were classified this way, yielding 728,818 true-positive and 1,695,534 true-negative pixels. Notably, the expert marked not all positive nuclei. The classified OHSC were taken from a previously published study (Grabiec et al., 2019).

#### Generation of Features Space

For quantification of neuronal damage with machine learning models, it was necessary to use features with strong discriminative power. As degenerated cells in our models were represented as comparably bright, circular structures, while background signals are more diffusely shaped with varying intensity, we used different spatial features highlighting this specific aspect.

Before feature generation, each image stack was normalized to the interval [0, 1] for comparability. Afterward, Gaussian derivatives were generated with a standard deviation σ of 1, 3, and 6 pixels and corresponding filter sizes depending on the order of the derivative as follows:


$$S\,i\,z\,e=\sigma *\left(3+0.25*o\,r\,d\,e\,r-\frac{2.5}{{\left(o\,r\,d\,e\,r-6\right)}^{2}+{\left(o\,r\,d\,e\,r-9\right)}^{2}}\right)$$

We used the first and second derivative in *x*, *y*, or *z* direction and the first and second derivative in *x* and *y* direction and *x*, *y*, and *z* direction. Additionally, Laguerre Gaussian functions (LGF) were used. LGF are defined as:


LrGFlp(x)=exp(-(ps)2/q)*(ps)l/q*

$${\left[\cos\left(l*\left\{\theta +\frac{2\,r\,\pi }{\max\left(2\,l,1\right)}\right\}\right)*L_{l}^{p}\,\left(p\,s*\text{arctan}\,\left(p\,s\right)\right)\right]}^{3-q}$$

With the Laguerre polynomials *L*. Here, we choose *s* = 0.5, *q* = 2, *r* = 0, *p* = 0, 1, 2, and *l* = 0, 1, 2. Notably, *r* = 0 was chosen as signals were point symmetrical and thus additional rotations of the LGF did not add significant information but would strongly increase the dimensionality of the feature space. Furthermore, Gaussian derivatives in the *x* and *y* direction or in the *z* direction of all LGFs were used as features. Additionally, LGF were applied to the coherency image. The coherency image *C* was obtained for each pixel using the eigenvalues λ of the structure tensor (Weichsel et al., 2010; Hohmann et al., 2020a):


$$C={\left(\frac{\lambda_{1}-\lambda_{2}}{\lambda_{1}+\lambda_{2}}\right)}^{2}$$

Further features were generated using top-hat transforms with sizes of 3, 4, 5, 7, 9, 13, 15, and 18 pixels and entropy filters of size 3, 5, 7, 9, 13, and 17 pixels. The filter size was set in such a way that the typical size of pycnotic nuclei, ranging from 5 to 15 pixels, was covered, including a small, additional margin at the lower and upper end. Taken together, 116 different features were generated. As some features were likely correlated, e.g., top-hat transforms of different sizes, a principal component analysis (PCA) was performed to de-correlate features and additionally reduce feature space. For all further steps, PCA components were used as features.

#### Feature Selection and Model Training and Testing

To reduce computational complexity, we first identified important features using a Wilcoxon rank sum test on the PCA components for true-negative against true-positive data. Afterward, features were sorted according to their *p*-value in ascending order. To test the importance of each feature, a 10-fold cross-validation was used with 30% of true-positive and true-negative data reserved for testing. The cross-validation was performed for all numbers of features, and the classification accuracy on the test set was evaluated to choose the optimal feature number. For analysis, four different types of machine learning algorithms were employed: NB, DA, RF, and MLP classifier. For the NB, a Gaussian distribution was used to model the data, and for the DA model, a quadratic discriminator was utilized. The RF classifier was trained with the following settings: 100 decision trees and a minimal leaf size of 100, and the interaction-curvature method was used to select the best split predictor. For the MLP model, a batch size of 5000, an adaptive learning rate with an initial learning rate of 0.1, and a L2 penalty (alpha) of 0.0001 were set. Model testing was performed in a leave-one-OHSC-out manner, training the models on the training data of all but one OHSC and classifying the remaining OHSC. For each OHSC, the number of true-positive, true-negative, false-positive, and false-negative pixels was recorded and used to calculate the classification accuracy.

#### Classification Into “High” and “Low” Neuronal Damage

As our training set contained a high variance in terms of neuronal damage and background levels, we additionally classified all images manually into either “high” or “low” damage and trained a support vector machine (SVM) to classify images using a bag of visual words as features. For assessing the goodness of this approach, a cross-validation step containing 70% of images for training and 30% for testing was performed, with 200 random divisions performed in such a way that the ratio of images with “high” damage to those with “low” damage was preserved. This step was performed in order to reduce variation between individual OHSC and improve classification. Both groups were otherwise handled as described before.

To test the hypothesis that splitting the data into “high” or “low” damage benefits classification accuracy, machine learning models were also trained on the whole image set for comparison.

#### Image Post-processing

To further reduce the number of false positives, several post-processing steps were included after the final prediction: First, all pixels with intensity lower than 0.1 times the maximal intensity of the current image were set as negatives; afterward, a morphological closing was performed and small objects (<30 pixels) were removed.

As a last step, another SVM was trained to differentiate between objects that are true and false positives, based on morphological features of each identified object. To identify objects as true or false positives, each object in the test set was associated with the manually assigned true-positive and true-negative objects. If the center of a predicted object was closer than 10 pixels to the center of an object that was manually marked as true-positive signal, the predicted object was considered true positive. In an identical fashion, false positives were identified. Predicted objects that did not match any of these criteria were discarded for training of the SVM, as they could not clearly be identified as true positives or false positives. From the objects identified this way, morphological parameters (area, convex area, eccentricity, equivalent diameter, extent, major axis length, minor axis length, perimeter, solidity, and circularity) were calculated and used as features for training. As model, a SVM with a radial base function as kernel and an assumed outlier fraction of 0.05 was used. For validation and estimation of prediction accuracy, a cross-validation with 30% of the objects used as test data was performed, with 200 random divisions performed in such a way that the ratio of true positives to false positives was preserved. These data were used to estimate the accuracy of differentiating between true- and false-positive objects in an image.

As a last post-processing step, the circular Hough transformation was applied to adjust for overlap of nuclei, taking advantage of the circular geometry of pycnotic nuclei, as reported before (Hohmann et al., 2018, 2020b). Therefore, each identified center point of the circles detected with the Hough transformation was matched with all manually assigned true-positive objects. If an object was closer than 10 pixels, it was assigned a true positive. To check whether this step improves the classification goodness, the number of true positives before and after Hough transformation was calculated to acquire a more accurate estimate of dead neurons. As not all true positives were labeled by the expert, only the recall was assessed (percent of correctly identified true positive objects).

### Statistics

Statistics was performed using a Wilcoxon rank sum test or a one-way ANOVA test with Tukey’s post-test. Significance was defined for *p* < 0.05.

## Results

### Feature Selection

**FIGURE 2:**
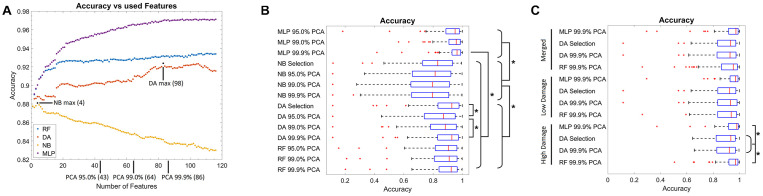
Training results. **(A)** Graph of the classification accuracy as a function of the defined features for the naïve Bayes, discriminant analysis, random forest, and multilayer perceptron classifier. Data points marked in black correspond to the optimal feature numbers for the naïve Bayes or discriminant analysis classifier. At the *x*-axis, the number of features necessary to explain 95, 99, and 99.9% of the variance in the feature data using principal components is marked. **(B)** Box plot of the classification accuracy of all models. The addition of “Selection” to the model name refers to the optimal feature number as extracted from **(A)**, while 95.0, 99.0, and 99.9% PCA refer to the feature number corresponding to the explained variance by the PCA components as marked in **(A)**. **(C)** Box plot of the classification accuracy when model training is performed on subsets of images corresponding to “low” and “high” neuronal damage. The “Merged” group corresponds to the merged data of the “low” and “high” neuronal damage classification results. Red lines in the box plot correspond to the median, blue boxes correspond to the 25th and 75th percentile, whiskers show the range of non-outlier values, and red “+” symbols show outliers. Stars depict statistically significant results with *p* < 0.05.

First, the optimal number of features for each model was evaluated (Figure 2A). Thereby, for the NB classifier, an optimal value of four features was found, and afterward, the in-sample classification accuracy declined steadily. For the DA, an optimal value of 98 features was identified, while for the RF and MLP, a plateau was reached when using 20 or 85 features, respectively. Notably, the classification accuracy was high for all models (>0.82). As the test data used for this step was in part also from the images used for training, it is likely overestimated (in-sample classification error). Thus, typical different feature numbers, based on the explained variance by the PCA components, were used in addition. Therefore, 4 features were used for NB, 98 for DA, and additionally 43, 64, and 86 features for all model types. These feature numbers correspond to the number of features necessary to explain 95, 99, or 99.9% of the variance of the data in terms of the PCA. For the RF and MLP, no further optimal feature number was selected, as the feature numbers taken *via* PCA represent an almost uniform sampling along the plateau.

### Model Testing

Using the abovementioned feature numbers, model accuracy was evaluated with a leave-one-OHSC-out strategy on all OHSC (Figure 2B). It thereby became apparent that classification results of the NB classifier were significantly inferior (median accuracies: 0.79–0.83) to the other three classifiers (median accuracies: 0.87–0.96, *p* < 0.05). Furthermore, the DA classifier using 86 or 98 features performed significantly better than the DA classifier using only 43 features (*p* < 0.05). For the RF classifier, no significant differences were observed when different numbers of features were compared. Notably, the MLP models using 86 features (median accuracy: 0.96) performed significantly better than any other non-MLP model (median accuracies: 0.79–0.92). Nevertheless, it has to be denoted that all models gave good to very good results, but some outliers with very low classification accuracy were found as well.

Based on the previous findings, only the DA classifier using 86 (99.9% PCA) and 98 (Selection) features, as well as the RF and MLP classifier using 86 (99.9% PCA) features were used for the next testing strategy. Based on the experts’ rating, OHSC were manually classified as either “high” (52 images) or “low” (65 images) damage. To automatize this step, a SVM was trained to differentiate between both classes. Using cross-validation, a median accuracy of 0.82 (standard deviation: 0.05) was estimated. Misclassifications mostly occurred on images of “intermediate” damage that could also not easily be classified by the expert.

Using these two subsets, the leave-one-OHSC-out test strategy was repeated using the selected models (Figure 2C). For the “high” damage subset, the RF (median accuracy: 0.97) and MLP model (median accuracy: 0.98) gave significantly better results than the DA models (median accuracies: 0.92–0.93; *p* < 0.05), while no statistically significant differences were found for the remaining groups. Furthermore, if the results of the “low” and “high” damage group are pooled together, the results using the RF classification (median accuracy: 0.95) approach significantly improved compared to the initial model trained on all OHSC (median accuracy: 0.92, *p* < 0.05).

### Identification of False-Positive Signals and Object Splitting

**FIGURE 3:**
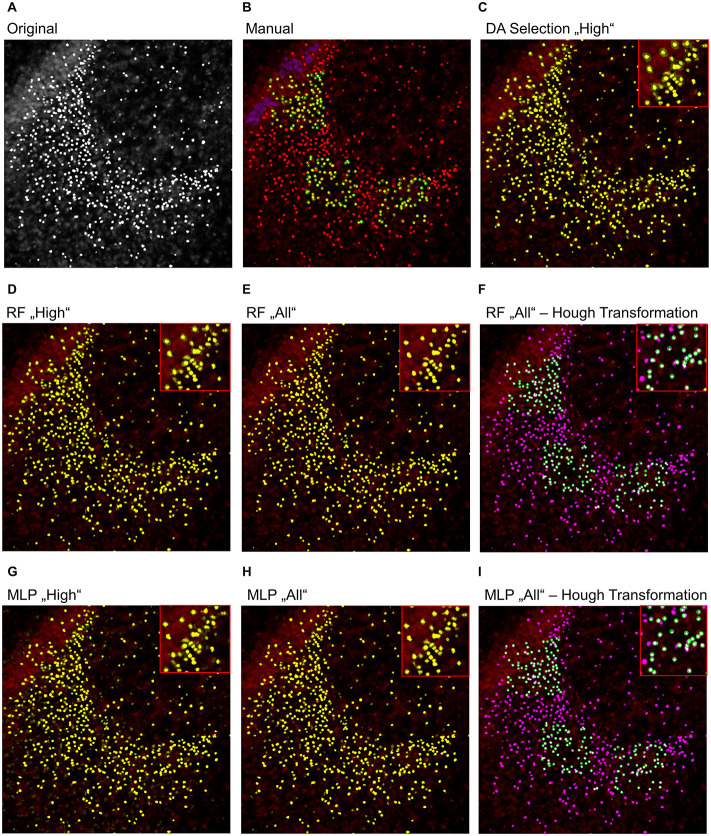
Example image of manual and automatic classification for images of the “high” damage class. **(A)** Original image, **(B)** manual classification, **(C)** discriminant analysis classifier trained on either “high” damage image subset, **(D)** random forest classifier trained on the “high” damage image subset, **(E)** the random forest classifier trained on all images, and **(F)** the identified objects, matched to the manually classified objects, **(G)** multilayer perceptron classifier trained on the “high” damage image subset, **(H)** the multilayer perceptron classifier trained on all images, and **(I)** the identified objects, matched to the manually classified objects for the multilayer perceptron classifier. Inlets depict magnifications of the corresponding image. **(B)** Yellow color corresponds to pixels classified as positive signals and purple corresponds to true negative signals. Green circles surround the points the expert clicked on. **(C–E,G,H)** Yellow color corresponds to pixels classified as positive signals. **(F,I)** Yellow corresponds to the manual classification, magenta to the automatic classification, white to an overlap of both, and green circles surround single objects, as identified by the Hough transformation that had a counterpart in the manual classification.

**FIGURE 4:**
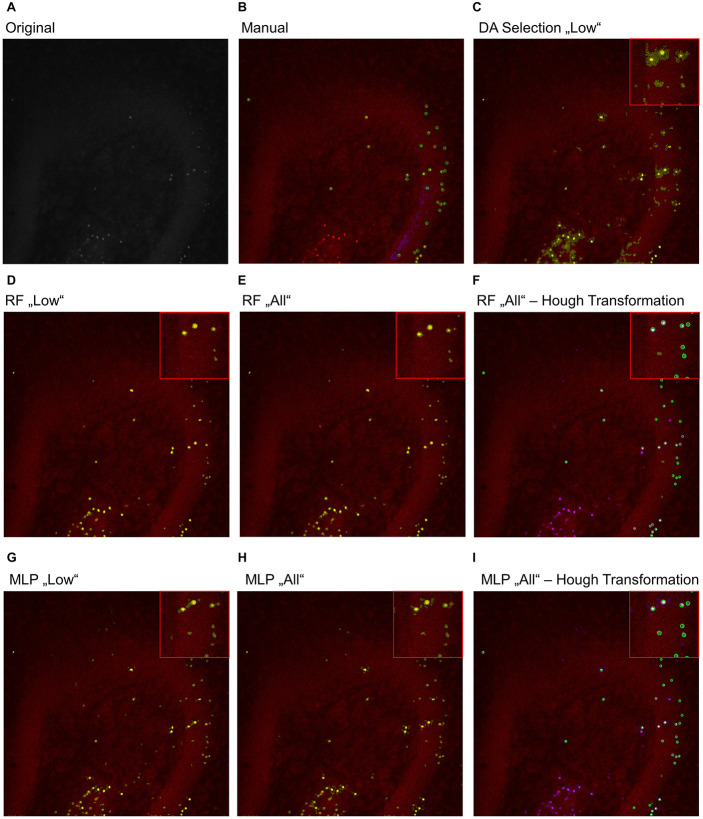
Example image of manual and automatic classification for images of the “low” damage class. **(A)** Original image, **(B)** manual classification, **(C)** discriminant analysis classifier trained on either “low” damage image subset, **(D)** random forest classifier trained on the “low” damage image subset, **(E)** the random forest classifier trained on all images and **(F)** the identified objects, matched to the manually classified objects, **(G)** multilayer perceptron classifier trained on the “low” damage image subset, **(H)** the multilayer perceptron classifier trained on all images, and **(I)** the identified objects, matched to the manually classified objects for the multilayer perceptron classifier. Inlets depict magnifications of the corresponding image. **(B)** Yellow color corresponds to pixels classified as positive signals and purple corresponds to true negative signals. Green circles surround the points the expert clicked on. **(C–E,G,H)** Yellow color corresponds to pixels classified as positive signals. **(F,I)** Yellow corresponds to the manual classification, magenta to the automatic classification, white to an overlap of both, and green circles surround single objects, as identified by the Hough transformation that had a counterpart in the manual classification.

To further analyze the classification accuracy of the proposed models, the DA, RF, and MLP classifiers were applied on the whole image set, instead of the manual classified subset of pixels (Figures 3, 4), as they performed best. Thereby, the DA was found to show over-segmentation for both the “high” and “low” damage classes (Figures 3C, 4C). This effect was significantly lower for the RF and MLP classifier trained on either the “high” or “low” damage subset of images or on all images (Figures 3D,E,G,H, 4D,E,G,H). From these images, it became apparent that some structures were incorrectly labeled as positives, especially for the DA classifier. Thus, the DA model was not analyzed further.

As the expert labeled both true-positive and true-negative signals, the opportunity was taken to match predicted objects in the final image with the ones manually classified. A total of 12,654 objects were considered as true positive and 178 as false positive when the RF model trained on all images was employed. On this basis, an SVM was trained on morphological features to differentiate between both classes to identify and remove false-positive objects. Using cross-validation, a median accuracy of 0.986 (standard deviation: 0.002) was calculated for differentiating between true and false positives. As the number of true-positive objects was approximately 71 times higher than those for false-positive objects, the sensitivity (correctly classified true positives) and specificity (correctly classified false positives) were additionally calculated. A median sensitivity of 0.9997 (standard deviation: 0.0007) and a median specificity of 0.055 (standard deviation: 0.021) were found.

For the MLP model, 12,504 true-positive and 274 false-positive objects were identified. Performing the same analysis, an accuracy of 0.978 (standard deviation: 0.002) was found for the SVM for discrimination between true and false positives with a sensitivity of 0.9989 (standard deviation: 0.0013) and specificity of 0.098 (standard deviation: 0.025).

Thus, while almost all true positives were correctly re-identified, only a small subset of false positives was found. Presumably, the very low number of false positives and thus training data mostly caused the low discriminative power, as the initial classification was already providing very good results. Nevertheless, the removal of false positives applying the trained SVM does provide small improvements.

As a last step, the Hough transformation was employed to separate overlapping pycnotic nuclei (Figures 3F,I, 4F,I). The application of the Hough transformation improved the classification clearly. Whereas initially 12,654 of 15,602 (81.1%) true-positive objects were correctly identified by using the RF model trained on all images, the value increased to 13,895 (89.1%) correctly classified objects after Hough transformation. For the MLP model, the recall of true-positive objects increased from 12,504 (80.1%) to 15,223 of 15,602 (97.6%) objects. While these numbers appear to strongly favor the MLP over the RF model, it has to be denoted that the MLP model also caused slightly more false-positive signals, as is, e.g., visible in Figures 3, 4.

## Discussion

The present study was conducted to develop a method and framework for the automatic assessment of neuronal damage in slice cultures. PI is frequently used to visualize the degenerating neurons and assess the extent of neuronal injury. Since measurement of PI fluorescence intensity did not show a positive correlation with the degree of neuronal damage when compared to methods counting dead neurons in dentate gyrus or cornu ammonis, new analysis approaches are needed (Ebrahimi et al., 2010). For assessing the validity of the here-introduced analysis scheme, we compared manual with automatic classification for different machine learning models. In general, the used models were found to provide good to very good results, with median pixel-wise accuracies up to 0.97 and an object recall of up to 97.6%, demonstrating the applicability of the presented approach.

Interestingly, the MLP and RF models were both performing very well, with slightly higher accuracies found for the MLP model. An exhaustive evaluation of different machine learning models found that, in general, RF models perform better than neuronal networks, including MLP (Fernández-Delgado et al., 2014). Yet, the authors of that study noted that neuronal networks tended to perform better for more complex data structures, making it tempting to speculate that this was the reason for the slightly higher accuracies observed for the MLP. Due to the complexity of both models (RF: 100 trees and MLP: 100 hidden layers), it cannot easily be concluded which parameters have been weighted in what way to explain the occurring differences.

### Comparison to Other Detection Approaches

Although several other automatic analysis approaches for the assessment of neuronal damage exist, a very common strategy is the manual counting of PI-positive, degenerated cells in the region of interest (Sun et al., 2009; Koch et al., 2011; Grabiec et al., 2019; Hohmann et al., 2019). Here, we focused on degenerating processes in the dentate gyrus of hippocampal slice cultures. The manual approach is not only time consuming but also subjective in terms of the definition of true-positive signals. While there is—to the authors’ knowledge—no study evaluating the reproducibility of manually counting pycnotic cells in PI-labeled brain slices, it was performed for analytically similar systems. For example, for the evaluation of γH2AX foci, which narrows down to counting bright dots with (partially) noisy background, it was shown that counting results depend on the rater (Herbert et al., 2014; Hohmann et al., 2018). Thus, the here-presented approach is a clear improvement, as it significantly speeds up the analysis process and is reproducible, if the trained model is provided. Nevertheless, as this machine learning approach imitates manual counting, it cannot provide a ground truth, as it is dependent on the training data and thus the rater.

Currently available (partly) automatic analysis approaches are all based on either purely measuring cumulative PI fluorescence intensity in a defined region of interest or segmentation of PI-positive nuclei using a manually set threshold (Dehghani et al., 2003; Hailer et al., 2005; Lee et al., 2011; Won et al., 2011; Pérez-Gómez and Tasker, 2012; Kim et al., 2014; Di Pietro et al., 2015; Masuch et al., 2016a; Grüßer et al., 2019). While the cumulative intensity measurement is objective, it showed no correlation to the manually counted number of dead neurons in OHSC (Ebrahimi et al., 2010). The noisy background with its region-dependent intensity might be the main reason for this finding. Notably, as the final models used here were trained on different OHSC with varying background levels, it is consequently able to handle different levels of background. The issue with manually selecting a threshold is its subjectivity and that the resulting signal is likely distorted by local variations in the signal-to-noise ratios, resulting in either over- or under-segmentation. To the authors’ knowledge, these aspects have not yet been investigated and studies are missing that compare the segmentation results to a manual ground truth. Consequently, quantifying the exact extent of these problems is challenging. Nevertheless, based on these issues, the presented approach seems promising as a multitude of features is used, covering a variety of morphological image features. Furthermore, false positives or overlap was not considered in any of the cited algorithms, while our analysis scheme provides an additional layer for detection of overlap and false-positive signals. Additionally, the provided scheme is not necessarily limited to PI labeling only, because the spatial features used for segmentation and morphological features for false-positive detections were used to identify approximately circular structures. Consequently, the models can be adapted to image modalities that narrow down to the detection of approximately circular objects in different settings.

Taken together, the presented set of algorithms clearly improves the existing approaches and can be used for reliably analyzing neuronal damage in PI-labeled slice cultures.

### Limitations

Despite the very good match between manual and automatic detections, the usage of the presented framework is limited in some aspects. For the analysis of dead cells in PI-labeled brain slices, the test data were split in a “low” and “high” damage class, used for training a SVM to assign OHSC to these classes. Thereby, it became evident that the trained SVM tends to misclassify OHSC of “intermediate” damage, leading to the comparably low accuracy of 0.82. Yet, these images were those that were also most difficult to assign for the expert. Consequently, it might be useful to introduce a third “intermediate” damage class for refining the system. Here, we did not introduce this damage class, as this would yield a significantly lower amount of training data for each class, making the resulting models less stable. For future studies trying to analyze PI uptake in brain slices, this more refined differentiation of damage classes is an option, but it has to be taken into account that the overall amount of training data needed will be significantly higher.

Another limitation lies within the usage of spatial features for assessing dead cells. As these features were used to take advantage of the specific morphology of pycnotic nuclei, they are adjusted to the resolution of the used images. Hence, the given approach is somewhat resolution dependent; thus, rescaling of images to a similar pixel size might be needed. Another issue is the variability of OHSC, as they can also be viewed in the presented classification results. As the amount of used training data was limited (117 OHSC), for some image modalities, only an insufficient amount of training data was available and thus classification accuracy was severely reduced for rare image modalities, corresponding to two to three OHSC of the whole training set, with accuracies below 0.6 for the MLP or RF model, respectively. For future studies to avoid such issues, a higher number of training data is needed to also adequately classify these images. Nevertheless, as these kinds of image modalities are rare (≈2% of OHSC), their impact is limited.

Similarly, the efficiency of the post-processing to identify false-positive signals was hampered, as only a very limited number of clearly identifiable false positives were available when using the final RF or MLP model, proving the robustness of the classification approaches. In future studies, this step needs further improvements, especially by providing more data on false positives. A last issue was the overlap of PI-positive nuclei. As only a semantic segmentation strategy was proposed, problems associated with overlap were not addressed, and thus, for images with high damage, an object splitting strategy had to be employed to avoid underestimation of neuronal damage. Based on previous studies in different fields, the Hough transformation was considered a useful and robust tool for object splitting, as PI-positive nuclei are circular (Hohmann et al., 2018, 2020b), but this approach is limited to roughly circular (or ellipsoid) shapes, and thus can only cover overlap to such a degree that single pycnotic nuclei still roughly resemble circles. Large accumulations of signals cannot be handled this way.

### Conclusion

In the present study, the applicability of spatial features for the classification of neuronal damage in PI-labeled slice cultures was tested, using multiple machine learning approaches. The used approaches led to very high classification accuracies up to 0.97, when using a MLP model. Additionally, a post-processing procedure was introduced to eliminate false-positive signals and handle signal overlap to improve classification results. The presented approach thereby appeared to be highly robust, with only few outliers in classification accuracy.

## Data Availability Statement

The raw data supporting the conclusions of this article will be made available by the authors, without undue reservation.

## Ethics Statement

The animal study was reviewed and approved by the Local Authorities for Care and Use of Laboratory Animals (State of Saxony-Anhalt, Germany, permission number: I11M18, date: 01.12.2012). Please note that the old permission date is the result of the evaluation of previously published data.

## Author Contributions

TH: conceptualization, software, and project administration. UH and TH: methodology, formal analysis, and visualization. UH: investigation. UH, FD, and TH: writing—original draft and writing—review and editing. FD and TH: supervision. All authors contributed to the article and approved the submitted version.

## Conflict of Interest

The authors declare that the research was conducted in the absence of any commercial or financial relationships that could be construed as a potential conflict of interest.

## Publisher’s Note

All claims expressed in this article are solely those of the authors and do not necessarily represent those of their affiliated organizations, or those of the publisher, the editors and the reviewers. Any product that may be evaluated in this article, or claim that may be made by its manufacturer, is not guaranteed or endorsed by the publisher.
