# Supplementary figures and images for: Assessment of Neuronal Damage in Brain Slice Cultures Using Machine Learning Based on Spatial Features
Source: Front Neurosci. 2021 Oct 8;15:740178. doi: 10.3389/fnins.2021.740178 (PMC8531652; doi:10.3389/fnins.2021.740178)

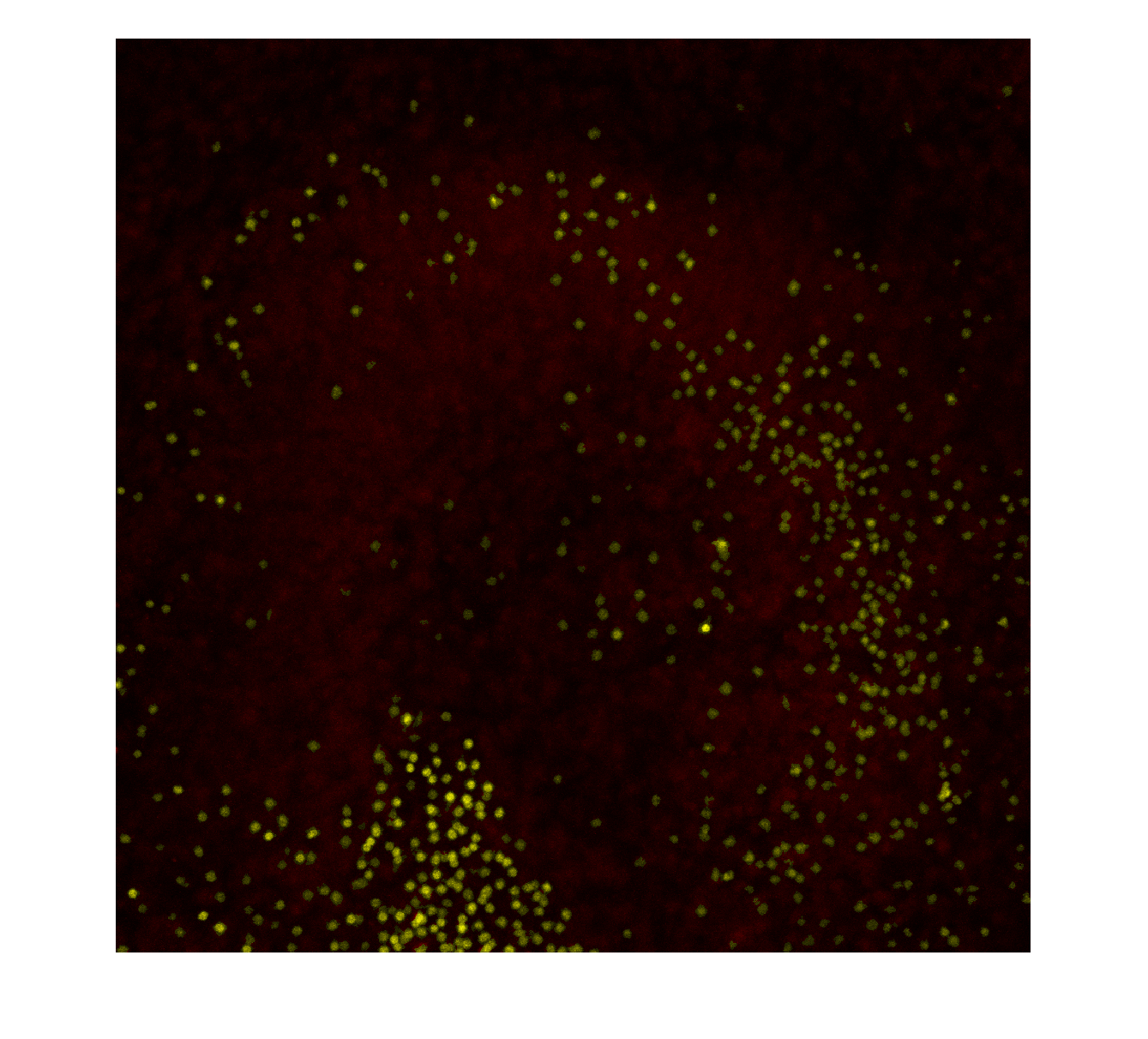

Supplement: Supplementary file 1 [file Data_Sheet_1.ZIP › SourceCode/Sample OHSC/3a_240114/3a_240114 TrainingData MLP All MinInt 0.1 ObjRemoval pca99.9% Overlay.png]

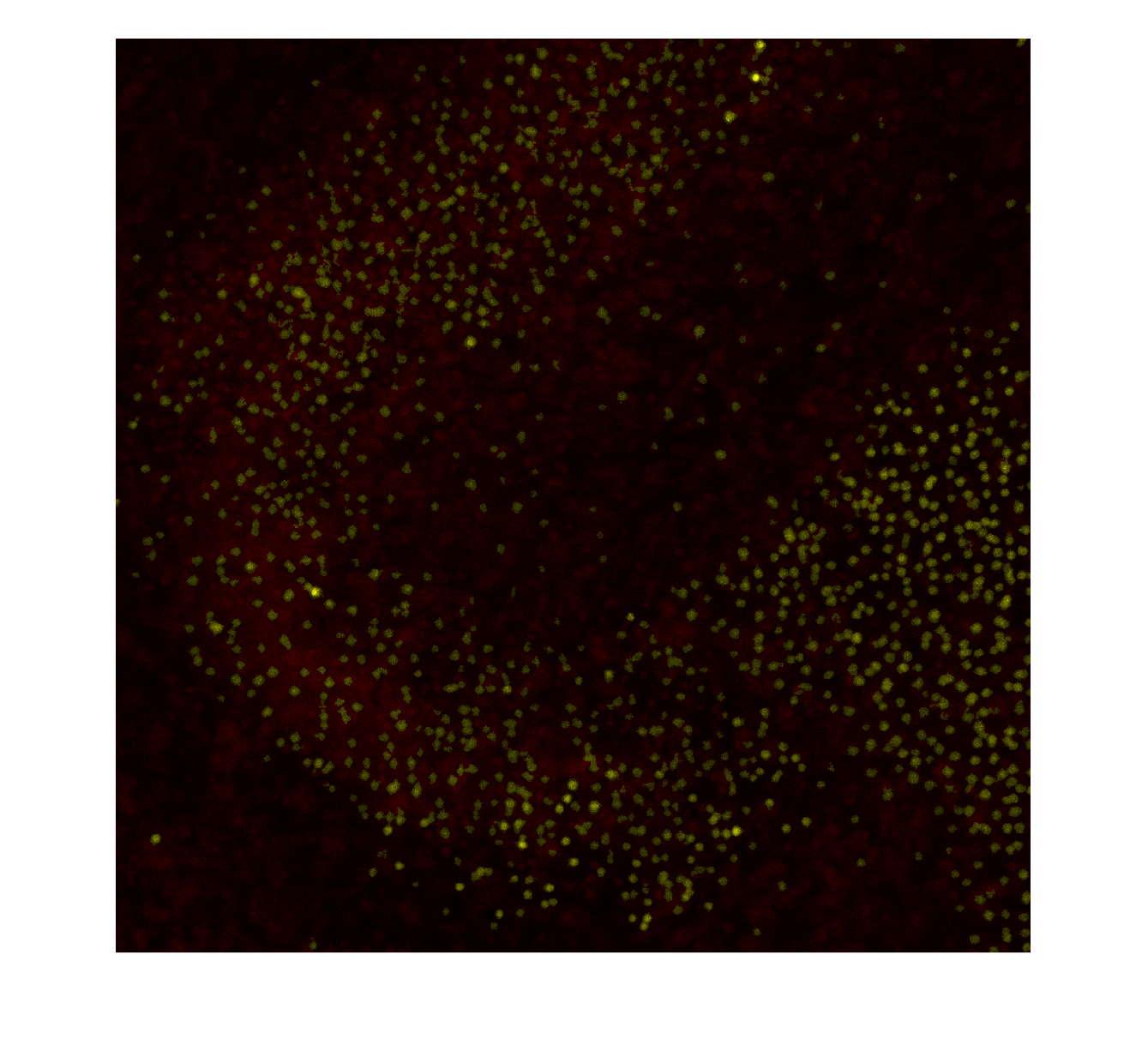

Supplement: Supplementary file 1 [file Data_Sheet_1.ZIP › SourceCode/Sample OHSC/3b_240114/3b_240114 TrainingData MLP All MinInt 0.1 ObjRemoval pca99.9% Overlay.png]

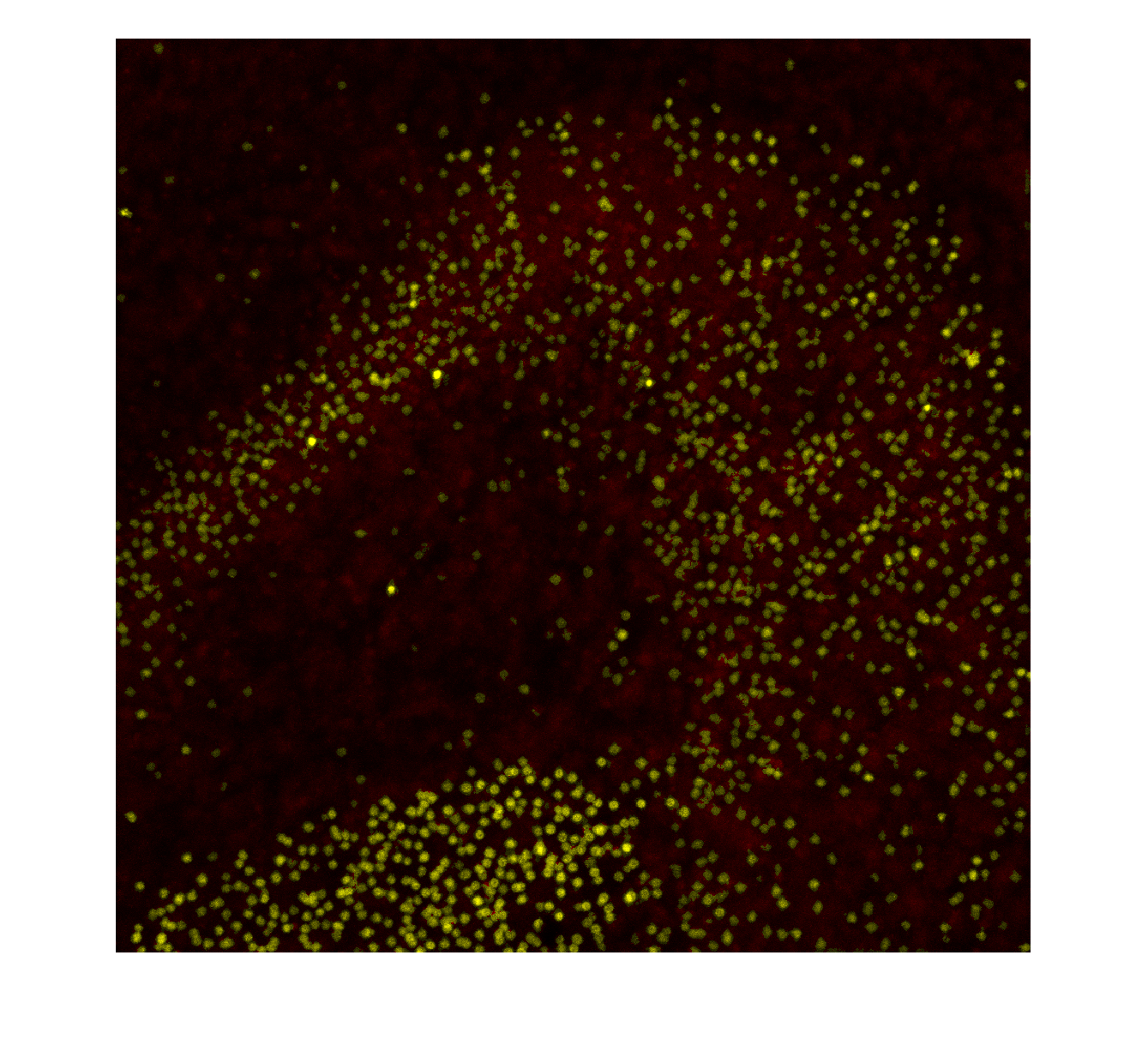

Supplement: Supplementary file 1 [file Data_Sheet_1.ZIP › SourceCode/Sample OHSC/3c_240114/3c_240114 TrainingData MLP All MinInt 0.1 ObjRemoval pca99.9% Overlay.png]
